# Reverse genetics in humanized mice reveals CARD8-mediated pyroptosis causing pancytopenia in human DPP9 deficiency

**DOI:** 10.1172/JCI207530

**Published:** 2026-09-15

**Authors:** Tianli Xiao, J. Richard Brewer, Maximillian Carlino, Ailin Han, Yamato J. Takabe, Chia-Yi Lee, Fengrui Zhang, Mi Chen, Holly Nicole Blackburn, Amin H. Nassar, Qiankun Wang, Kristen Brennand, Liang Shan, Esen Sefik, Diane S. Krause, Richard A. Flavell

**Affiliations:** 1 Department of Immunobiology,; 2 Howard Hughes Medical Institute, and; 3 Yale Stem Cell Center, Yale School of Medicine, New Haven, Connecticut, USA.; 4 Department of Pathology and Molecular Medicine, Yale University, New Haven, Connecticut, USA.; 5 Department Jof Genetics, Yale University School of Medicine, New Haven, Connecticut, USA.; 6 Department of Laboratory Medicine, Yale University, New Haven, Connecticut, USA.; 7 Department of Surgery, Yale School of Medicine, New Haven, Connecticut, USA.; 8 Division of Medical Oncology, Yale Cancer Center, New Haven, Connecticut, USA.; 9 Institute of Infectious Diseases, Shenzhen Bay Laboratory, Shenzhen, Guangdong, China.; 10 Department of Psychiatry, Division of Molecular Psychiatry, Yale University School of Medicine, New Haven, Connecticut, USA.; 11 Institute of Human Immunology, Shenzhen Medical Academy of Research and Translation, Shenzhen, China.; 12 Department of Cell Biology, Yale School of Medicine, New Haven, Connecticut, USA.

**Keywords:** Hematology, Immunology, Hematopoietic stem cells, Innate immunity

## Abstract

Loss-of-function mutation in the human gene dipeptidyl peptidase 9 (*DPP9*) causes Hatipoglu syndrome leading to severe inflammasomopathy. A key feature of the disease is pancytopenia, and patients require bone marrow transplantation, but the mechanism of cell loss is unclear, since *Dpp9*-mutant mice have normal hematopoiesis, suggesting that a distinct mechanism of disease occurs in humans. Here, we present a model of human DPP9 deficiency leveraging reverse genetics in the MISTRG6 humanized mice. We found that CRISPR editing of human CD34^+^ hematopoietic stem and progenitor cells (HSPCs) led to very efficient and persistent gene deletion in vivo. Human *DPP9* deletion recapitulated cytopenia in peripheral blood and in the bone marrow, and cell loss was cell intrinsic. However, *DPP9* deletion led to few transcriptional changes suggesting posttranscriptional regulation in human HSPCs. Mechanistically, DPP9 deficiency led to activation of the CARD8 inflammasome, resulting in HSPC pyroptosis, whereas NLRP1 was dispensable for cell death. Thus, our results reveal a unique human mechanism of disease and offer therapeutic insight for this inflammasomopathy.

## Abstract 2

We develop a new way to model human genetic disease and discover why patients with DPP9 deficiency have loss of blood cells.

## Introduction

Rare autoinflammatory syndromes arising from dysregulation of the inflammasome, or inflammasomopathies, have revealed important mechanisms of human innate immunity. Although many of the principles in inflammasome biology are shared between mice and humans, over 75 million years of distinct microbial exposure have imposed different evolutionary pressure on inflammasome sensors, raising the question of whether some inflammasomopathies have distinct mechanisms between humans and model organisms such as mice.

Recently, an inflammasomopathy caused by loss-of-function mutations in the dipeptidyl peptidase 9 (*DPP9*) locus was identified in several families (1, 2). These patients present with recurrent fevers, repeated infections, pancytopenia, and anemia, and elevated serum levels of inflammatory cytokines were reported in 1 patient. Current treatment requires bone marrow transplantations (1). How human *DPP9* mutations cause pancytopenia remains an important question, since pancytopenia probably causes recurrent infection due to the lack of immune cells and the need for bone marrow transplantation. Strikingly, this hematopoietic loss is not recapitulated in mice — *Dpp9* mutants in conventional mouse models maintain normal immune cell numbers, and their hematopoietic stem cells (HSCs) retain full reconstitution capacity upon serial transplantation (1, 3, 4). Rather than dismissing this discrepancy as a limitation of animal modeling, we reasoned that the divergent evolutionary trajectories of mouse and human inflammasome biology may have endowed human HSCs with a distinct, species-specific vulnerability, one that, if identified, could illuminate not only the pathogenesis of DPP9 deficiency, but a previously unrecognized axis of human blood stem cell regulation.

*DPP9* encodes dipeptidyl peptidase 9, which typically cleaves X-P dipeptides, where X is any amino acid. It also serves as the endogenous inhibitor of mouse nucleotide-binding domain (NBD)-containing, leucine-rich repeat (LRR)-containing and pyrin domain-containing protein 1 (NLRP1), human NLRP1, and human caspase recruitment domain-containing protein 8 (CARD8) inflammasomes (5–10). NLRP1 and CARD8 are 2 structurally related inflammasomes that cause pyroptotic cell death and release IL-1β and IL-18 proinflammatory cytokines upon activation (7, 8, 11–14). Both NLRP1 and CARD8 contain a regulatory N-terminus and a cytotoxic C-terminus. The cytotoxic C-terminal peptide contains a CARD domain that can interact with ASC or caspase 1 (CASP1) for inflammasome activation. However, spurious activation by these C-terminal peptides is prevented by DPP9 through the formation of a ternary complex (5, 6). During inflammasome activation, the N-terminal peptide undergoes “functional degradation” by the proteasome, which releases additional cytotoxic C-terminal fragments and overwhelms DPP9 complexes, leading to inflammasome activation (15, 16). Importantly, NLRP1 and CARD8 are poorly conserved between mice and humans to the point that human and mouse NLRP1 are activated by distinct signals (17, 18). On the other hand, the CARD8 inflammasome is absent in most rodent species including *Mus musculus* but is widely expressed in human hematopoietic cells (18, 19).

We address how human DPP9 deficiency causes pancytopenia in vivo by leveraging the MISTRG6 (see Methods for definition) humanized mouse model. MISTRG6 mice enable the engraftment of human CD34^+^ hematopoietic stem and progenitor cells (HSPCs) by modification of several key genes to express human macrophage–CSF (M-CSF) (20, 21), IL-3, and granulocyte-macrophage–CSF (GM-CSF) (22), signal regulatory protein alpha (SIRPα) (23), thrombopoietin (24), and IL-6 (21, 25) on a *Rag^–/–^* and *IL2rg^–/–^* background (26, 27). Physiologic expression of these human factors is achieved by gene-knockin replacement of their murine counterparts. The immunodeficient background, combined with expression of human factors, enables human CD34^+^ HSPCs to engraft and differentiate into a near-complete complement of the human immune system, including human NK cells, monocytes, and macrophages, T cells, and B cells. Human HSCs persist long term in the bone marrow of MISTRG6 mice and can be serially transplanted up to 4 generations (26–28). f and a related family of strains have revealed mechanisms of human immunity in the context of infection, cancer, autoimmunity, and hematopoiesis (26, 28–39).

To resolve this species-specific paradox, we combined xenotransplantation, CRISPR gene editing, flow cytometry, CFU assays, competitive transfers, and single-cell transcriptomics and found that human *DPP9^–/–^* HSPCs underwent pyroptosis through activation of the CARD8 inflammasome, which triggered CASP1-dependent cell death. Deletion of *CARD8* or *CASP1* rescued cytopenia and loss of DPP9-deficient bone marrow stem cells, whereas NLRP1 deficiency did not. Given that *CARD8* is absent from the mouse genome yet highly enriched in human hematopoietic cell populations, we propose that the mouse-human discrepancy in DPP9 deficiency reflects a fundamentally distinct inflammasome logic governing human blood stem cell survival, one in which CARD8 serves as a sentinel whose unchecked activation is sufficient to collapse human hematopoiesis.

## Results

### Efficient and persistent gene KO of human CD34^+^ HSPCs in the MISTRG6 humanized mouse.

**Figure 1:**
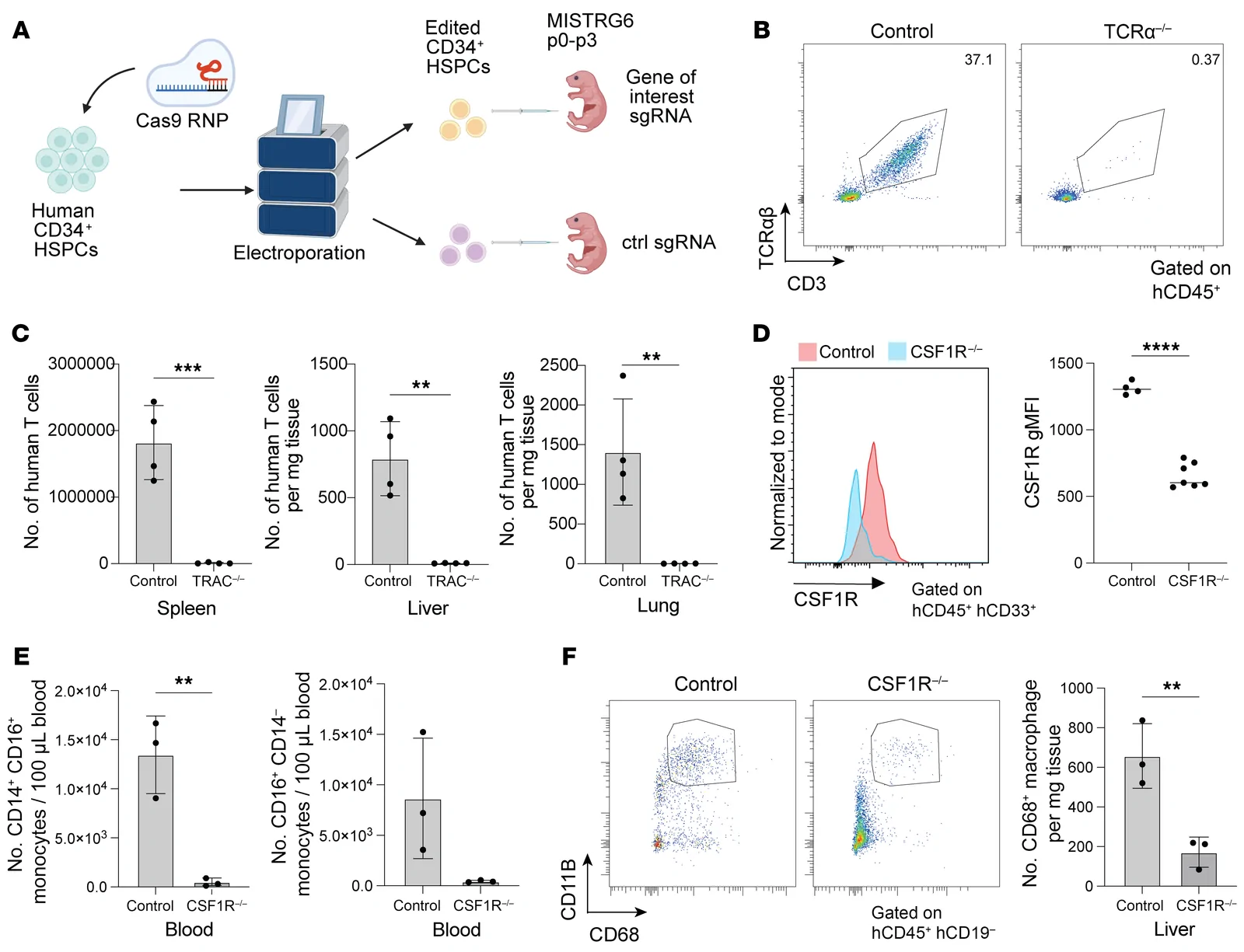
Efficient and persistent gene KO in human CD34^+^ HSPCs in the MISTRG6 humanized mouse model. (**A**) Schematic of gene editing followed by engraftment in the MISTRG6 humanized mouse model. MISTRG6 pups were injected intrahepatically in the first 3 days after birth without preconditioning. (**B** and **C**) *TRAC^–/–^* and control CD34^+^ HSPCs were engrafted into MISTRG6 mice, and the mice were assessed 12-16 weeks after engraftment. Data were pooled from 2 experiments. (**B**) Representative flow plot from splenic cells. (**C**) Number of human T cells in tissues. (**D**–**F**) *CSF1R^–/–^* and control CD34^+^ HSPCs were engrafted into MISTRG6 mice and assessed 9 weeks after engraftment. (**D**) Expression of CSF1R on human CD33^+^ myeloid cells in the blood 9 weeks after engraftment. Representative histogram and summary data are shown. (**E**) Number of CD14^+^CD16^+^ and CD16^+^CD14^–^ monocytes in the blood. (**F**) Number of CD68^+^ macrophages in the liver. Experiments are representative of results from mice engrafted with cells from 2 human donors. **P ≤ 0.01, ***P ≤ 0.001, and ****P ≤ 0.0001, by 2-tailed Student’s *t* test. Data shown as mean ± SD.

Since DPP9 deficiency has different phenotypes in mice and humans, we set out to establish a model of human DPP9 deficiency using the MISTRG6 humanized mouse system. To generate DPP9 deficiency or gene KO in general, we initially tested the feasibility of generating CD34^+^ HSPCs from induced pluripotent stem cells (iPSCs) using a protocol from Stemcell Technologies. However, iPSC-derived CD34^+^ cells failed to generate substantial (>1%) engraftment in MISTRG6 mice, despite expressing human HSC markers such as CD34, CD90, and CD49f (Supplemental Figure 1, A and B; supplemental material available online with this article; https://doi.org/10.1172/JCI207530DS1). Therefore, we explored gene editing in primary human CD34^+^ HSPCs isolated from cord blood or fetal liver. We optimized the ratio and quantity of Cas9 and sgRNA to delete T cell receptor alpha constant (*TRAC*) encoding the T cell receptor α chain (TCR-α), which is required for T cell development, in human CD34^+^ HSPCs. Inference of CRISPR edits analysis (40) revealed that CRISPR ribonucleoproteins (RNPs) generated large truncations in the *TRAC* locus (Supplemental Figure 1, C–E). We further optimized culture conditions to promote CD34^+^ HSPC recovery following gene editing (Supplemental Figure 1F). Next, we assessed whether edited cells could engraft in the MISTRG6 animals and whether gene KOs persisted in vivo. Immediately after electroporation, we administered edited CD34^+^ HSPCs or control cells, which were edited with control sgRNA RNPs, via intrahepatic injection into neonatal MISTRG6 pups (Figure 1A) without preconditioning. Deletion of *TRAC* was persistent in vivo, as mice engrafted with *TRAC^–/–^* human HSPCs failed to generate human T cells in the spleen, liver, or lung up to 16 weeks after engraftment, whereas control mice had abundant human T cells (Figure 1, B and C). Development of other human hematopoietic cells was not impaired, thus preserving B cells and myeloid cells (Supplemental Figure 1, G–I). As an additional proof of concept, we tested deletion of human *CSF1R*. As expected, CSF1R expression was significantly decreased in human blood monocytes in MISTRG6 mice 9 weeks after engraftment (Figure 1D). Furthermore, the number of CD16^+^ monocytes and liver macrophages were significantly decreased in the blood (Figure 1, E and F). MISTRG6 mice with *CSF1R^–/–^* HSPCs retained lymphoid cell development, and human CD45^+^ cells were similar in *CSF1R^–/–^* HSPCs (Supplemental Figure 1, J and K). Taken together, CRISPR RNPs led to highly efficient KO in human CD34^+^ HSPCs that persisted in vivo in MISTRG6 humanized mice.

### Human DPP9 deficiency leads to pancytopenia and bone marrow failure.

**Figure 2:**
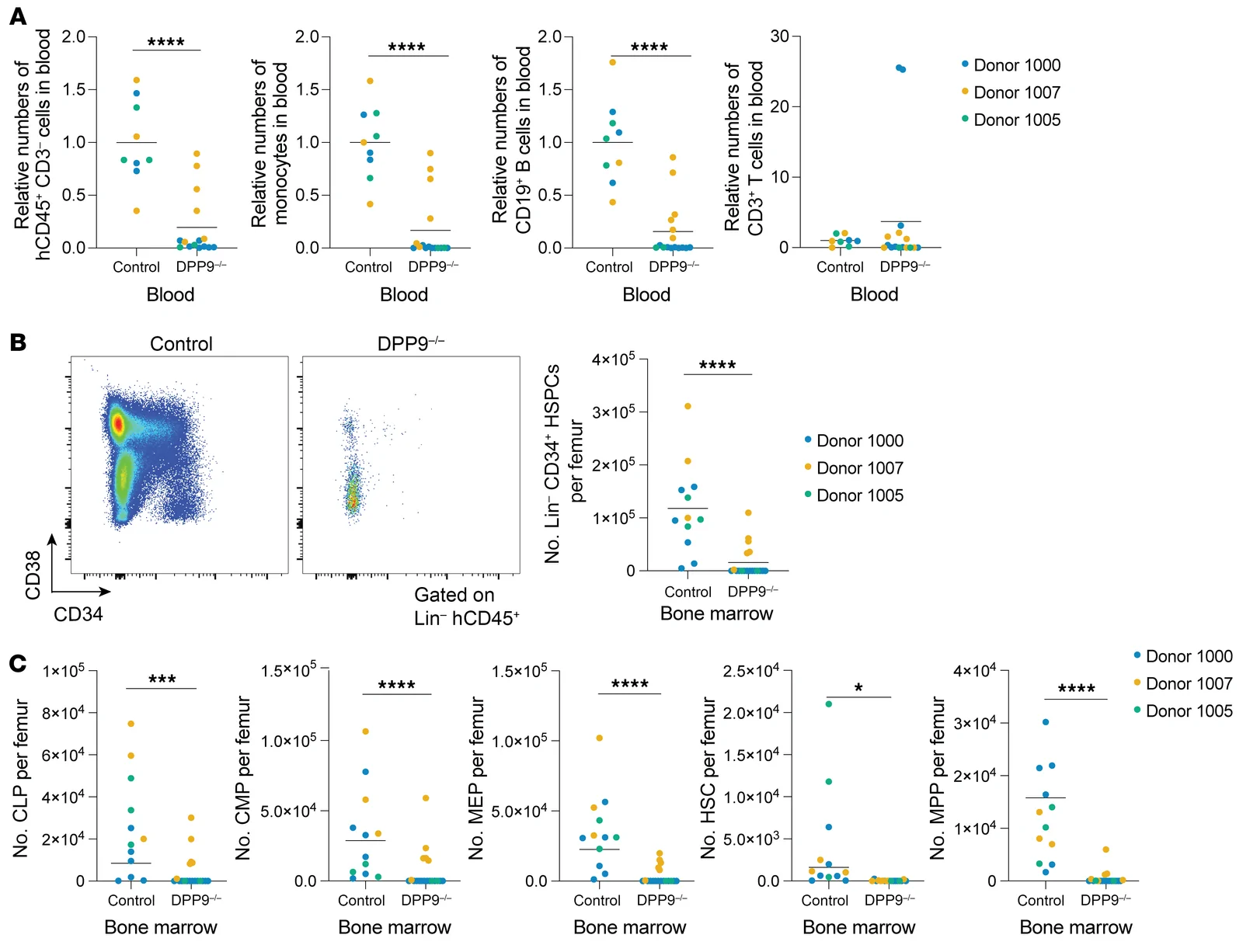
Human DPP9 deficiency results in cytopenia and loss of bone marrow HSPCs. MISTRG6 mice were engrafted with control or *DPP9^–/–^* CD34^+^ HSPCs for 8–9 weeks. (**A**) Relative number of cells in the blood. hCD45, human CD45. (**B**) Representative flow plot and summary data of lineage^–^CD34^+^ HSPCs in the bone marrow. (**C**) Number of CD34^+^ HSPC subsets in the bone marrow. Relative cell numbers were calculated by normalizing cell counts of each animal to the mean value of the control group per donor and per engraftment into MISTRG6 mice. *P ≤ 0.05, ***P ≤ 0.001, and ****P ≤ 0.0001, by 2-way ANOVA between genotypes. Data are shown as grand mean.

To assess the consequence of human DPP9 deficiency in vivo, we designed 3 sgRNAs targeting the catalytic exon of *DPP9* in CD34^+^ HSPCs and engrafted them via intrahepatic injection into MISTRG6 mice, and then assessed their engraftment 8–9 weeks later. PCR amplification of the KO region revealed efficient deletion in vitro and in vivo after engraftment (Supplemental Figure 2A). Mice engrafted with *DPP9^–/–^* HSPCs (hereafter referred to as *DPP9^–/–^* mice) had significantly fewer human CD45^+^ cells in the blood, including monocytes and B cells (Figure 2A), thus recapitulating the leukopenia seen in patients with *DPP9* germline mutations (1). T cell numbers were comparable between control and *DPP9^–/–^* mice, likely because once an initial wave of T cell progenitors differentiate, undergo homeostatic expansion and become activated, they become resistant to CARD8-mediated cell death. Once T cells become activated, they become resistant to CARD8-mediated cell death (41, 42). Furthermore, in many humanized mouse models, T cells preferentially expand in poorly engrafted mice and thus do not faithfully reflect stem cell differentiation in the bone marrow (43). The phenotype was consistent between human donors (Figure 2A). We reasoned that peripheral cytopenia may be due to a loss of stem cells in the bone marrow. Indeed, *DPP9^–/–^* Lin^–^CD34^+^ HSPCs failed to persist in the bone marrow (Figure 2B). Further dissection of the CD34^+^ cell population revealed that the number of common lymphoid progenitors (CLPs), common myeloid progenitors (CMPs), megakaryocyte-erythroid progenitors (MEPs), HSCs, and multipotent progenitors (MPPs) were significantly reduced in *DPP9^–/–^* mice (Supplemental Figure 2B and Figure 2C). To ensure that loss of *DPP9^–/–^* HSPCs was not due to their inability to home to the bone marrow, we assessed whether direct intrafemoral injection of *DPP9^–/–^* HSPCs could ameliorate engraftment in vivo. Despite direct access to bone marrow space, *DPP9^–/–^* HSPCs failed to persist in the bone marrow, and cytopenia was still observed in the blood in those animals (Supplemental Figure 2C).

### Loss of CD34^+^ DPP9^–/–^ HSPCs is cell intrinsic.

**Figure 3:**
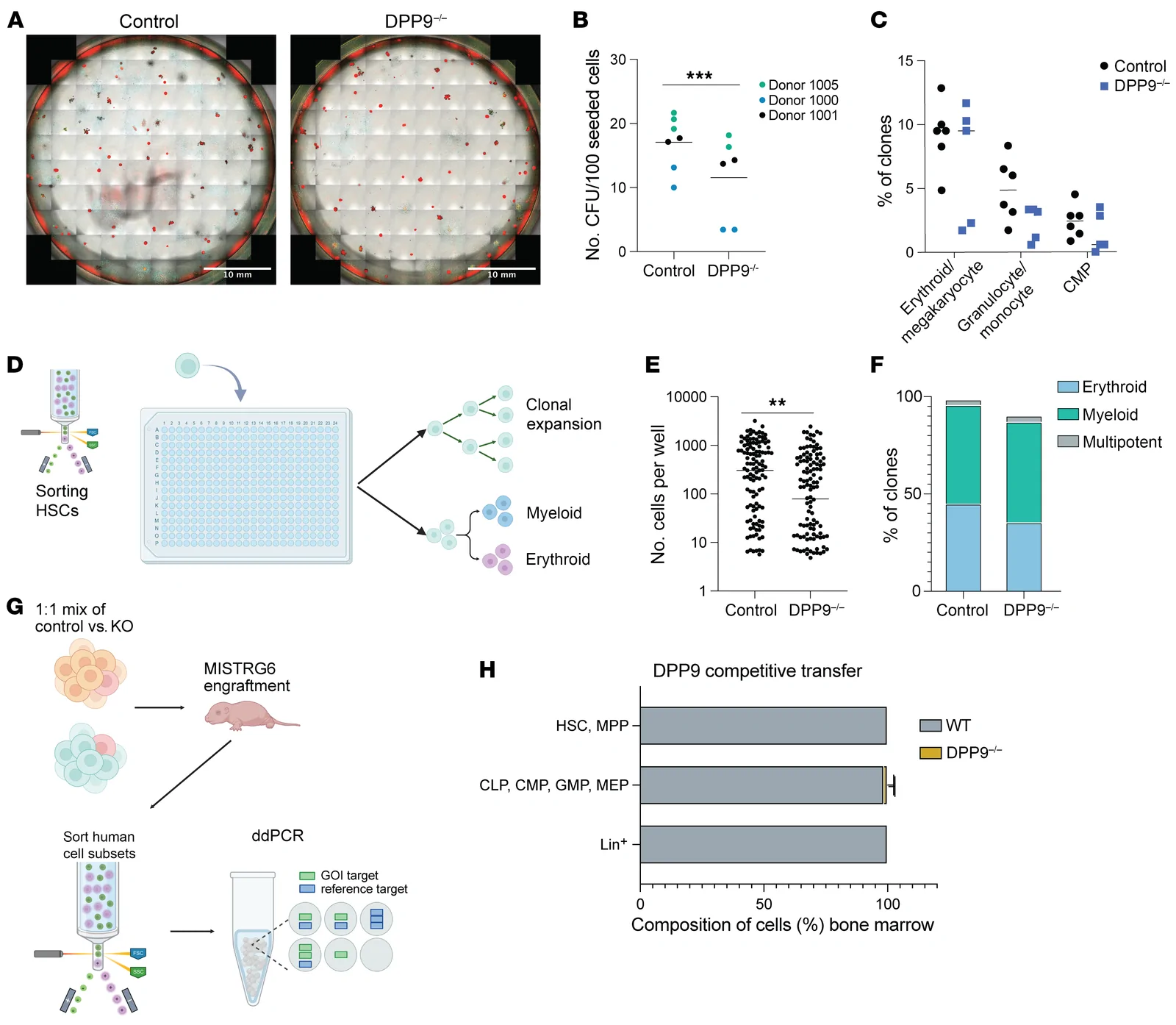
Loss of CD34^+^ *DPP9^–/–^* HSPCs is cell intrinsic. (**A**) In vitro CFU assay plate. Red: CD235a; yellow: CD66b; cyan: CD14; green: CD41. Scale bars: 10 mm. (**B**) Summary of the in vitro CFU assay. (**C**) Lineage differentiation from the in vitro CFU assay. (**D**) Schematic of the sorting of single hCD45^+^CD34^+^Lin^–^CD38^–^CD90^+^ HSCs into a 384-well plate for in vitro expansion and a differentiation assay. (**E**) Number of cells per well after a 7-day expansion of sorted HSCs. (**F**) Differentiation toward myeloid (CD66b^+^ or CD14^+^) and erythroid (CD235a^+^) lineages from sorted HSCs. Clones that gave rise to both fates are labeled as multipotent. (**G**) Schematic of the competitive transfer experiment using MISTRG6 mice. (**H**) Composition of human bone marrow cells 7 weeks after competitive transfer. **P ≤ 0.01 and ***P ≤ 0.001, by 2-tailed Student’s *t* test. Data shown as grand mean (**B**), mean (**C**), mean ± SD (**H**), median (**E**).

To assess whether loss of *DPP9^–/–^* HSPCs is recapitulated in vitro, we knocked out DPP9 and cultured control (*TRAC^–/–^*) and *DPP9^–/–^* HSPCs in liquid culture. Surprisingly, *DPP9^–/–^* HSPCs expanded normally in vitro (Supplemental Figure 3A). We reasoned that defects with DPP9 mutants would be more prominent at a clonal level, thus we seeded control and *DPP9^–/–^* CD34^+^ cells in a CFU assay utilizing MegaCult medium supplemented with cytokines supportive of granulocyte, monocyte, megakaryocyte, and erythroid lineages to detect any specific lineage biases (Figure 3, A–C). We observed fewer colonies formed by *DPP9^–/–^* HSPCs (Figure 3B), although the effect was much more modest than loss of HSPCs in vivo. DPP9 mutation did not lead to lineage bias (Figure 3C), consistent with our findings in vivo that all lineages were affected by DPP9 mutation (Figure 2C). To specifically assess the effect of DPP9 deficiency on HSCs, we sorted single CD34^+^CD38^–^CD90^+^ human HSCs into a 384-well plate and cultured them with either an expansion medium or differentiation medium (Figure 3D). Similar to bulk CD34^+^ cell populations, *DPP9^–/–^* HSCs expanded to fewer cells at the end of the 7-day expansion (Figure 3E), and the percentage of clones that had substantial expansion (>100 cells) was also lower in *DPP9^–/–^* HSCs (Supplemental Figure 3B). In contrast, differentiation into myeloid and erythroid lineages was similar between control and *DPP9^–/–^* HSCs (Figure 3F). Finally, to determine whether loss of *DPP9^–/–^* HSPCs in vivo is cell intrinsic, we engrafted a 1:1 mixture of control and *DPP9^–/–^* HSPCs in MISTRG6 mice and assessed the frequency of *DPP9^–/–^* HSPCs after 7 weeks (Figure 3G). Unlike competitive transfer assays in conventional mouse models, in which each donor is congenically marked, control and *DPP9^–/–^* HSPCs from the same human donor would not express different cell-surface markers. To distinguish the cells derived from the 2 genotypes, we leveraged digital droplet PCR (ddPCR) to detect the fraction of control and KO cells (39, 44–48). Thus, we sorted various bone marrow cell populations from the competitive transfer recipient mice and found that *DPP9^–/–^* cells were nearly completely lost in the HSCs, progenitor cells, and differentiated Lin^+^ cells (Figure 3H). These data suggest that loss of *DPP9^–/–^* occurred in a cell-intrinsic manner.

### DPP9 deficiency leads to few transcriptional changes in HSPCs.

**Figure 4:**
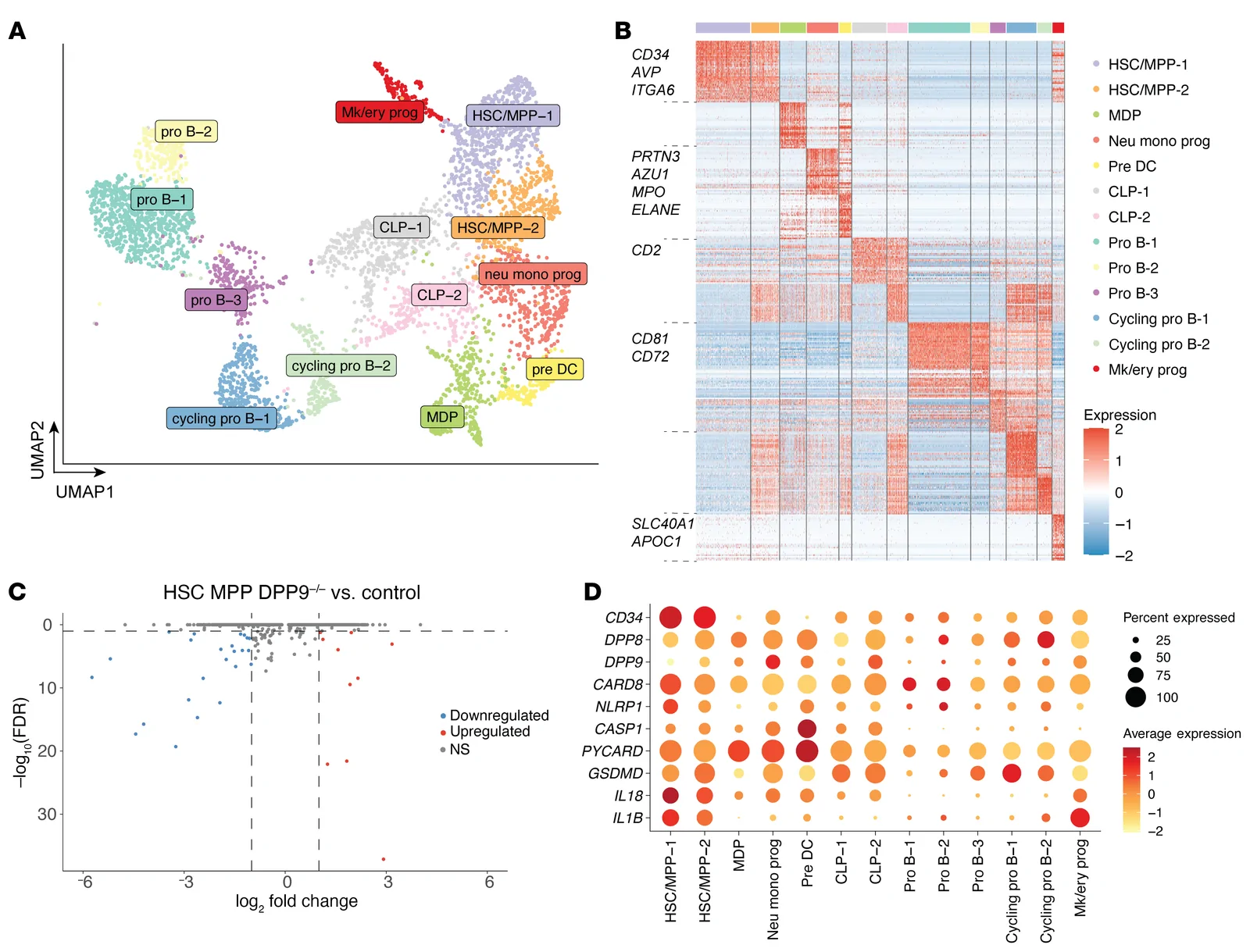
DPP9 deficiency in hCD34^+^ HSPCs does not result in drastic transcriptional changes. scRNA-seq analysis of sorted human Lin^–^CD34^+^ cells from the bone marrow of MISTRG6 mice engrafted with either control or *DPP9^–/–^* CD34^+^ HSPCs, 4 weeks after engraftment. (**A**) Heterogeneity of human Lin^–^CD34^+^ cells visualized by UMAP. (**B**) Heatmap of diagnostic genes of each cluster from the UMAP in **A**. The top-30 genes are plotted for ease of visualization. (**C**) Volcano plot of DE genes between *DPP9^–/–^* and control cells from HSC–MPP-1 and HSC–MPP-2 clusters. (**D**) Gene expression of various inflammasome components across various human stem and progenitor subsets. CD34 expression (top) serves as a reference of a highly expressed gene.

As a preliminary step to mechanistically understand how *DPP9^–/–^* HSPCs are lost in vivo, we performed single-cell RNA-seq (scRNA-seq). We generated both control and *DPP9^–/–^* HSPCs from 2 human donors and engrafted littermate MISTRG6 animals as above. Four weeks after engraftment at a point when some *DPP9^–/–^* cells were still present, we sorted Lin^–^CD34^+^ cells. Control and *DPP9^–/–^* HSPCs were separately hashtagged and encapsulated together. After demultiplexing, we obtained robust scRNA-seq data from over 5,000 cells, with an average of 5,000 genes per cell. Dimensional reduction using uniform manifold approximation and projection (UMAP) and cluster analysis revealed 13 distinct clusters (Figure 4A). To identify them, we iteratively overlaid the signature from a large human bone marrow dataset (49) onto our clusters and identified 2 HSC-MPP clusters, 3 myeloid progenitor clusters, 2 CLP clusters, 5 lymphoid progenitor clusters, and 1 megakaryocyte-erythroid progenitor cluster (Figure 4A and Supplemental Figure 4A). We confirmed that each cluster expressed specific markers of their population (Figure 4B and Supplemental Table 1). For example, the HSC-MPP clusters expressed high levels of *CD34*, *AVP*, and *ITGA6* (encoding CD49f), which are canonical markers of human HSCs (Supplemental Figure 4B). Similarly, CLPs expressed *CD2*, whereas the pro–B cell progenitors expressed *CD81* and *CD72*. On the other hand, myeloid progenitors expressed *PRTN3*, *AZU1*, *MPO*, and *ELANE*. Megakaryocyte and erythroid progenitors expressed *SLC40A1* and *APOC1* (Figure 4B). Notably, the frequency of *DPP9^–/–^* cells was relatively decreased in the HSC-MPP clusters, consistent with their loss (Supplemental Figure 4C).

To assess transcriptional changes in *DPP9^–/–^* cells, we performed differential expression analysis between control and *DPP9^–/–^* cells. We combined clusters of similar identity together to reveal potential subtle differences between genotypes, and combined the 2 HSC-MPP clusters (HSC–MPP-1, HSC–MPP-2), the 3 myeloid progenitor clusters (monocyte-dendritic cell progenitor, neutrophil monocyte progenitor, pre- DC), the 2 CLP clusters (CLP-1, CLP-2), and pro–B cell clusters (pro–B-1, pro–B-2, pro–B-3). Since DPP9 deficiency led to a loss of HSCs in vivo (Figure 2C), we focused our analysis on the HSC-MPP clusters. We detected only 77 differentially expressed (DE) genes in HSCs and MPPs (Figure 4C, Supplemental Figure 4C, and Supplemental Table 1). Ingenuity Pathway Analysis revealed that several signaling pathways were downregulated in *DPP9^–/–^* HSCs and MPPs, which was primarily due to downregulation of *FOS* and *JUN*. Similarly, we found only 29 DE genes in myeloid progenitor cells, 14 DE genes in CLPs, and 16 DE genes in pro–B cell clusters (Supplemental Figure 4E and Supplemental Table 1). Taken together, scRNA-seq revealed that DPP9 deficiency did not lead to substantial transcriptional changes across any hematopoietic progenitor cell populations, indicating that DPP9 controlled HSPC fate primarily through posttranscriptional mechanisms. DPP9’s function as a protein-level inhibitor of the CARD8 and NLRP1 inflammasomes raises the possibility that unrestrained inflammasome activity, rather than any transcriptional reprogramming, underlies the progressive loss of DPP9-deficient HSPCs. To evaluate this, we first asked whether the relevant inflammasome machinery is even present in human HSPCs during xenotransplantation. Strikingly, *CARD8* was broadly and robustly expressed across CD34^+^ progenitor subsets, while *NLRP1* expression, though detectable, was considerably lower. Downstream effectors including *CASP1* and *GSDMD* were similarly expressed, indicating that human HSPCs possess the complete molecular apparatus required for inflammasome-driven pyroptosis (Figure 4D). *CARD8*’s absence in the mouse genome combined with the species-specific nature of the hematopoietic defect, pointed directly to CARD8 as the candidate mediator of DPP9-deficient HSPC loss and prompted us to test this genetically.

### CARD8 mediates pyroptosis of DPP9^–/–^ HSPCs.

**Figure 5:**
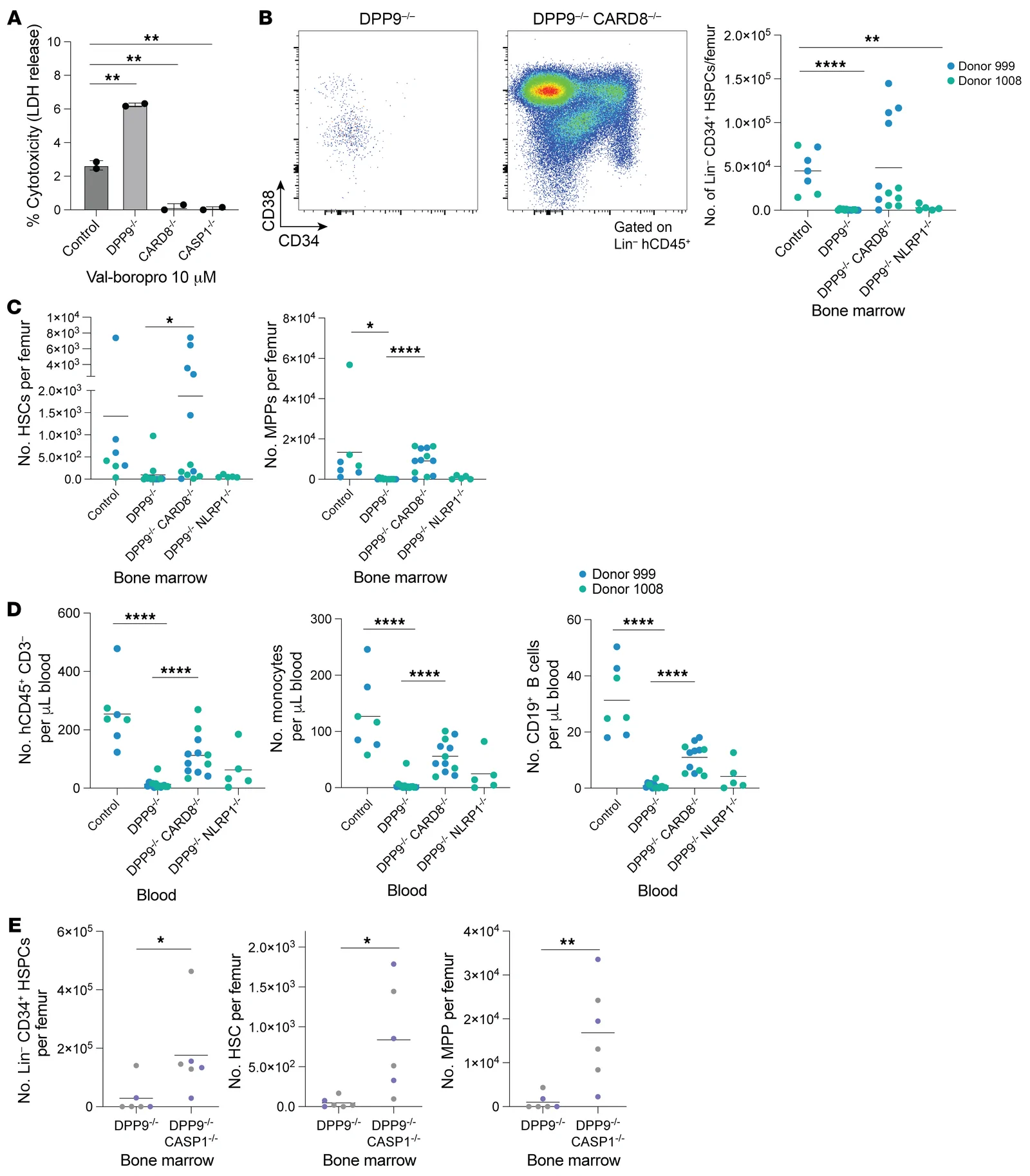
CARD8 mediates the loss of *DPP9^–/–^* HSPCs. (**A**) Human CD34^+^ HSPCs were treated with VbP for 20 hours, and LDH was measured. (**B**–**D**) MISTRG6 mice were engrafted for 10–11 weeks. Cells from bone marrow (**B** and **C**) and blood (**D**) were quantified. (**E**) MISTRG6 mice were engrafted for 9–11 weeks. *P ≤ 0.05, **P ≤ 0.01, and ****P ≤ 0.0001, by 2-tailed Student’s *t* test. Data shown as mean ± SD (**A**), grand mean (panel **B**–**E**).

To determine whether DPP9 deficiency leads to CARD8-mediated pyroptosis in CD34^+^ HSPCs, we cultured edited CD34^+^ HSPCs for 3 days and stimulated them with Val-boropro (VbP), an inhibitor of DPP8 and DPP9 (7, 50). VbP treatment led to HSPC pyroptosis, as measured by lactate dehydrogenase (LDH) release. Notably, VbP elicited an even higher degree of pyroptosis in *DPP9^–/–^* HSPCs, suggesting that DPP8 also plays a role in sequestering the inflammasome from activation. Pyroptosis was completely abrogated in HSPCs that lacked *CARD8* or *CASP1*, suggesting that HSPCs undergo CARD8-dependent pyroptosis (Figure 5A). To determine whether CARD8 mediated loss of *DPP9^–/–^* HSPCs in vivo, we performed double-KO experiments to determine whether loss of CARD8 rescues *DPP9^–/–^* HSPCs. We found that simultaneous gene KOs led to high KO efficiency without marked loss of cell numbers (Supplemental Figure 5, B and C). We engrafted control, *DPP9^–/–^*, *DPP9^–/–^ CARD8^–/–^*, and *DPP9^–/–^ NLRP1^–/–^* HSPCs from the same donor into littermate MISTRG6 mice. As expected, *DPP9^–/–^* HSPCs were lost after engraftment. HSPC numbers were rescued by deletion of *CARD8*, suggesting that CARD8 mediated pyroptosis of *DPP9^–/–^* HSPCs in vivo, including HSCs and MPPs (Figure 5, B and C). Interestingly, *NLRP1* deletion failed to rescue *DPP9^–/–^* HSPCs. (Figure 5, B and C). Leukopenia in the blood was also prevented, as the number of human CD45^+^ cells, including monocytes and B cells, recovered after deletion of *CARD8* but not *NLRP1* (Figure 5D). To examine whether *DPP9^–/–^* HSPCs underwent pyroptosis, we deleted *CASP1*, which also rescued the loss of *DPP9^–/–^* HSPCs (Figure 5E). Taken together, these results reveal that *DPP9^–/–^* HSPCs undergo CARD8-mediated pyroptosis after transplantation and that loss of bone marrow stem cells leads to peripheral cytopenia.

## Discussion

The absence of a hematopoietic phenotype in conventional mouse models of *Dpp9* mutations contrasts with the devastating pancytopenia observed in patients. *Dpp9*-mutant mouse HSCs were normal, since competitive transfer of WT and *Dpp9*-mutant fetal liver cells leads to equal reconstitution in recipient mice and after secondary transplantation (3, 4). Additionally, the presence or absence of the mouse NLRP1 inflammasome did not alter the number of bone marrow leukocytes in *Dpp9*-mutant mice (1). Rather than reflecting a failure of the mouse model, we argue that this discrepancy implies that the human hematopoietic compartment acquired a distinct inflammasome-dependent vulnerability. *DPP9* mutations in patients lead to enzymatic hypomorphs, decreased protein expression, or premature translation stoppage (1, 2), which we modeled in human HSPCs by generating a gene KO targeting the *DPP9* catalytic exon. Our experiments revealed multilineage loss in the bone marrow and blood. The recapitulation of patient disease and the cell-intrinsic loss of stem cells suggest that the susceptibility to pyroptosis was a property of HSPCs rather than a consequence of transplantation models. The near-absence of transcriptional changes in DPP9-deficient HSPCs further reinforced that DPP9 operated principally as a posttranscriptional gatekeeper. Our results indicate that DPP9 sets the activation threshold of the CARD8 inflammasome in human blood stem cells and, when this regulation is disrupted, triggers their depletion.

The hyperactivation of the CARD8 inflammasome in human HSPCs in our in vivo model aligns with prior in vitro experiments, which revealed increased NLRP1 and CARD8 activation in cells carrying *DPP9* mutations found in patients (1, 2). However, the selective requirement for CARD8, rather than NLRP1, in mediating the death of DPP9-deficient HSPCs remains poorly understood. One possibility is that human DPP8 was sufficient to prevent NLRP1, but not CARD8, activation in *DPP9^–/–^* HSPCs. Indeed, in human keratinocytes, knockdown of both DPP8 and DPP9 is required for efficient NLRP1 activation (10). A second possibility is that *CARD8* is preferentially expressed in human HSPCs, unlike *NLRP1*, which is highly expressed in barrier tissue cells in humans (17, 51, 52). Indeed, we found that *CARD8* was well expressed in various stem and progenitor cell populations, whereas *NLRP1* was transcriptionally expressed at a lower level. Finally, a third possibility is that CARD8 is activated in the bone marrow microenvironment. Distinct and shared stressors activate human NLRP1 and CARD8 inflammasomes. Specifically, human NLRP1 is activated by ribotoxic stress (13, 14, 53, 54), viral RNA (55–57), toxins (58, 59), and viral proteases (15, 60–63). Human CARD8 is activated by various viral proteases, notably the HIV protease, which induces pyroptosis in human T cells (11, 12, 19, 41). Recently, both NLRP1 and CARD8 were described to be activated by proteotoxic stress and reductive stress (50, 64–67). CARD8 may be triggered by a yet-to-be-defined cellular stress signal during transplantation of HSPCs and induce their pyroptosis in the absence of DPP9. How cell types determine whether to activate the NLRP1 or CARD8 inflammasome will require further study. We also noted that the loss of *DPP9^–/–^* human HSPCs was far more pronounced in vivo than in vitro. This disparity may have stemmed from differing experimental timeframes — over 7 weeks in vivo versus less than 2 weeks in vitro — or it could suggest that in vivo signals actively accelerated *DPP9^–/–^* HSPC loss. For example, stem cells may compete for limited quantities of survival and expansion signals, such as human thrombopoietin (THPO) within the bone marrow niche. They may also accumulate proliferation-induced protein folding stress capable of triggering CARD8 activation, as discussed above (50). Overall, the phenotypic differences in vivo and in vitro reinforce the value of using in vivo humanized mouse models to study human stem cell regulation.

Unlike human *NLRP1*, whose gain-of-function mutations cause skin pathology (68) reflective of its high expression in barrier cells, gain-of-function mutations of mouse *Nlrp1* cause pyroptosis of macrophage progenitor and granulocyte-macrophage progenitors (GMPs). These mice have decreased numbers of lymphoid, myeloid, and erythroid cells in addition to systemic inflammation driven by IL-1β and IL-18 (69). Notably, mouse *Nlrp1* is highly expressed in hematopoietic lineages (69), akin to the expression pattern of CARD8 in humans. Thus, human CARD8 and mouse NLRP1 may share some functional similarity, although each inflammasome may be distinctly regulated, owing to structure and sequence differences between these sensors (18).

Beyond DPP9 biology, our study establishes a generalizable platform for interrogating human-specific gene function in vivo. By generating control and KO cells from the same human donor and using littermate MISTRG6 mice as recipients, we avoided the confounding effects of diverse human genetics between donors and isolated the role of specific human genes in vivo (26, 70, 71). Thus, this approach can be applied to understand the role of other human genes that are poorly modeled in conventional mouse models. Given that SNP variants of *DPP9* were recently identified to regulate outcomes of idiopathic pulmonary fibrosis and SARSCoV2 (72–74), our study highlights the role of human DPP9 in restraining CARD8 inflammasome activation in human hematopoietic cells and contributes to our understanding of this emerging axis in human inflammatory diseases.

## Methods

### Sex as a biological variable.

Both male and female human CD34^+^ donor cells and both male and female recipient MISTRG6 mice were used in this study. Similar findings are reported for both sexes.

### Mice and engraftment.

MISTRG6 mice are previously described (21, 25, 26). Briefly, mice were generated by combining strains made by the R. Flavell laboratory, the M. Manz laboratory, and Regeneron Pharmaceuticals, based on a *Rag2*^−/−^ *IL2rg*^−/−^ 129 x BALB/c background, followed by additional gene knockin of human *CSF1*, *IL3/CSF2*, *SIRPA*, *THPO*, and *IL6* in their respective mouse loci (21, 25, 26). Experimental mice were obtained by crossing MITRG6 mice with MISTRG6 mice to obtain MIS^h/m^TRG6 mice, such that the mice were heterozygous for mouse and human SIRPα, and M-CSF, IL-3/GM-CSF, thrombopoietin, and mice were homozygous for the M-CSF, IL-3/GM-CSF, thrombopoietin, and IL-6 human genes. For simplicity, these MIS^h/m^TRG6 recipients are referred to as MISTRG6 mice in all other sections of this manuscript. For engraftment, newborn MIS^h/m^TRG6 mice between 1 and 3 days of age were injected intrahepatically with 30,000 purified CD34^+^ human HSPCs resuspended in 20 μL using a 31 gauge insulin syringe (BD). Neonatal MIS^h/m^TRG6 mice were not preconditioned prior to engraftment. Engrafted pups were cross-fostered by CD1 dams until weaning. CD1 Elite mice were purchased from Charles River Laboratories. Both male and female mice were used for engraftment. For intrafemoral engraftment, adult MIS^h/m^TRG6 mice were sublethally irradiated at 1.5 Gy (x-ray with the X-RAD 320 irradiator). Mice were anesthetized under isoflurane, and a hole was made in the femur, followed by injection of 30,000 CD34^+^ HSPCs. The appropriate littermate controls were always used. All animals were housed in specific pathogen–free facilities at the Yale Animal Resource Center.

### Isolation of CD34^+^ HSPCs.

CD34^+^ cells were isolated from fetal liver purchased from Celce (previously known as Advanced Bioscience Resources) or from cord blood collected by the Yale University Reproductive Sciences Biobank. For CD34^+^ HSPC isolation from fetal liver, liver tissue was mechanically dissociated into small pieces and digested stirring in 10% FBS in RPMI containing 1 mg/mL collagenase D and 0.06 mg/mL DNAse I for 20 minutes. Hepatocytes were removed by centrifugation at 50*g*, and CD34-enriched cells were obtained through density-gradient centrifugation with Lymphocyte Separation Medium (PromoCell) at 1,000*g* without brake for 20 minutes. Finally, CD34^+^ HSPCs were isolated by positive selection using the EasySep Human CD34 Positive Selection Kit II (Stemcell Technologies) following the manufacturer’s protocol. CD34^+^ HSPC isolation from cord blood was performed using the EasySep Human Cord Blood CD34 Positive Selection Kit II (Stemcell Technologies) following the manufacturer’s protocol. Briefly, HSPCs were enriched by adding the RosetteSep cocktail to whole blood followed by centrifugation on a LymphoPrep (Stemcell Technologies) density gradient. Enriched mononuclear cells then underwent positive selection to purify CD34^+^ HSPCs. HSPCs were stored in 10% DMSO in FBS in liquid nitrogen.

### CRISPR editing.

CD34^+^ human HSPCs were used immediately after isolation or recovered from frozen vials by culturing overnight in SFEMII medium (Stemcell Technologies) supplemented with 100 ng/mL human SCF, 100 ng/mL human TPO, 100 ng/mL human FLT3L, 20 ng/mL human IL-6 (cytokines from Peprotech), 0.75 μM StemRegenin 1 (Cayman Chemical), 500 nM UM729 (Stemcell Technologies) at a density of 500,000 cells/mL. CRISPR RNP was generated by combining 40 pmol Cas9 (IDT), 100 pmol sgRNA (Synthego), and PBS in 4 μL for 10 minutes at room temperature. RNP is stable in 4°C. Since 2 or 3 guides are used per gene, a mix of sgRNA totaling 100 pmol was used per reaction. HSPCs were concentrated by centrifugation and resuspended in 20 μL P3 buffer (Lonza, V4XP-3032), combined with CRISPR RNP and electroporated using the Lonza 4D Nucleofector using the DZ-100 program and p3 buffer. Cells were rested in warm medium for 30 minutes before engraftment into recipient MISTRG6 mice. To assess the editing efficacy by Inference of CRISPR Edit Analysis (40), DNA from edited cells was extracted using an H_2_O solution containing 50 mM Tris, 1 mM EDTA, and 0.5% Tween-20 supplemented with 0.6 mg/mL proteinase K incubated at 55°C for 3 hours or overnight and heat inactivated at 95°C. Alternatively, DNA was extracted using the Quick-DNA MicroPrep kit (Zymo Research). A region flanking the sgRNA cut sites was amplified by PCR and submitted for analysis on the EditCo website (https://ice.editco.bio/). The guide sequences were as follows: AAVS1 control-1, GGGGCCACUAGGGACAGGA; AAVS1 control-2, CCGGCCCUGGGAAUAUAAGG; TRAC-1, CUCUCAGCUGGUACACGGCA; TRAC-2, GAGAAUCAAAAUCGGUGAAU; TRAC-3, ACAAAACUGUGCUAGACAUG; CSF1R-1, CCAGGGCGAGAAGGAGUAGU; CSF1R-2, CACCUUUCUGCACUUUCAGC; CSF1R-3, CAAUGCAGUGCCCUGAUGGG; DPP9-1, GGAUUAGCCCCAUGAGCGAG; DPP9-2, GGUGGAGAUCGAGGACCAGG; DPP9-3, CAUGGAUGGCAACUCGGCUC; CARD8-1, UAAGAUAGGACACGAGGUAA; CARD8-2, GACACGGCAGAGCAAGAAUG; CARD8-3, CCGGCAACUCCAAGCCAGGA; NLRP1-1, GAUAGCCCGAGUGACAUCGG; NLRP1-2, CAGAGUUCCAUAAUGAGGUG; NLRP1-3, GUUCAGCUUGAGCCAGUCCU; NLRP1-4, UUUCAGGAGGACUCCCAAGG; NLRP1-5, UAUGUGAUGCAGCUCCACCC; NLRP1-6, UCCCAAGUGACUGCUCCAUU; CASP1-1, UUUAUCCGUUCCAUGGGUGA; CASP1-2, CUAAACAGACAAGGUCCUGA.

### iPSC cultures.

Human iPSC cell lines were derived and reported in a previous study (75). Human iPSCs were grown as sparse aggregates in mTeSR plus media (Stemcell Technologies) and differentiated into hematopoietic progenitor cells (HPCs) using the STEMdiff hematopoietic kit (Stemcell Technologies) following the manufacturer’s protocol. Briefly, iPSC aggregates larger than 50 μm were seeded at a density of 10–20 aggregates/cm^2^ in a 12-well plate. Aggregates were cultured in base medium supplemented with medium A for 3 days, and a half-medium change was performed on day 2. Cells were cultured in base medium supplemented with medium B from day 3 to day 12, with a half-medium change performed on days 5, 7, and 10. If needed, cells were stored in CS10 freezing media (Stemcell Technologies) prior to in vivo engraftment.

### Cell isolation for flow cytometry and cell sorting.

MISTRG6 mice were euthanized with 100% isoflurane. Blood was collected retro-orbitally into EDTA-containing solution for a final concentration of 4 μM EDTA. Blood was directly stained with antibodies on ice. After washing, RBC lysis and fixation were achieved in 1 step using the RBC Lysis/Fixation Solution (BioLegend) following the manufacturer’s protocol. For cell isolation from the bone marrow, femurs were isolated using dissection tools and crushed using a mortar and pestle, and then filtered through a 70 μm filter. For cell isolation from the liver, liver was perfused. Immune cells were isolated using RPMI with 2% FBS and 0.25 mg/mL collagenase II (Gibco, Thermo Fisher Scientific), 0.1 mg/mL DNAse I (grade ii, 10104159001, Roche), and 5 mM calcium (MilliporeSigma). The same liver lobe was chopped into small pieces using scissors and digested, stirring at 37°C for 30 minutes at 400 rpm. Enzymes were quenched by adding 40 mL media, and hepatocytes were removed by centrifugation at 100*g* for 5 minutes, 2 times. Immune cells from the supernatant were isolated through centrifugation at 500*g* for 5 minutes. RBCs were removed through ACK lysis. For cell isolation from the lung, the lung was digested with RPMI supplemented with 2% FBS, 1 mg/mL collagenase D (MilliporeSigma), and 0.1 mg/mL of grade II DNAse I (Roche). Surface antigen staining was performed as previously described (76). Briefly, single-cell suspensions were stained with live/dead stain in PBS on ice for 10 minutes and then stained for cell-surface antigens followed by washing in PBS supplemented with 2% FBS. When appropriate, cells were fixed with 2% paraformaldehyde in PBS until analysis. Cell sorting was performed on the BD FACSAria or Bigfoot Spectral Cell Sorter. Flow cytometric analysis was performed using BD Symphony or Cytek Aurora.

The following antibodies were used (all from Biolegend): anti–human CD3 (OKT3); anti–human CD10 (HI10a); anti–human CD14 (HCD14); anti–human CD16 (3G8); anti–human CD19 (HIB19); anti–human CD20 (2H7); anti–human CD33 (WM53); anti–human CD34 (clone 561); anti–human CD38 (HIT2); anti–mouse CD45 (30-F11); anti–human CD45 (HI30); anti–human CD45RA (HI100); anti–human CD49f (GoH3); anti–human CD56 (HCD56); anti–human CD66b (G10F5); anti–human CD90 (5E10); anti–human CD123 (6H6); anti–human CD68 (Y1/82A); and anti–human HLADR (L243). Live/Dead fixable viability dye yellow (Thermo Fisher Scientific) distinguished live and dead cells.

### ddPCR.

DNA from cells were isolated using the Quick-DNA MicroPrep kit (Zymo Research). ddPCR was performed according to the manufacturer’s protocol (Bio-Rad). Briefly, 25 μL ddPCR Master Mix was prepared with 2X ddPCR Super Mix for probes (no deoxyuridine triphosphate), 900 nM primer, and 250 nM hexachlorofluorescein or 6-carboxyfluorescein probes. Droplets were generated from the quantitative PCR (qPCR) master mix using the QX200 droplet generator (Bio-Rad). PCR was then performed on the C1000 Touch Thermal Cycler (Bio-Rad). The fluorescence of each droplet was read using the QX200 Droplet Reader (Bio-Rad). To determine the fraction of KO and control cells, a single DNA sample was fractionated into more than 20,000 droplets, and 2 PCR reactions were performed within each droplet. One PCR reaction with HEX fluorescent signal amplified a reference *DPP9* sequence that was present in both control and *DPP9^–/–^* cells. The other PCR reaction (with FAM fluorescent signal) amplified the sgRNA-targeted DPP9 sequence targeted by the *DPP9* sgRNA, such that control cells, but not *DPP9^–/–^* cells, would have PCR amplification. An absolute quantification of DNA copies for the *DPP9* reference and KO region was determined by quantifying the number of FAM^+^ or HEX^+^ droplets. Control cells had an equal quantity of reference and KO amplifications, whereas *DPP9^–/–^* cells had only reference amplifications. By comparing the ratio of reference and KO amplification, we could quantify the presence of control and *DPP9^–/–^* cells.

### CFU assays and in vitro expansion assays.

The CFU assay was performed as previously described (77). CD34^+^ cells were electroporated as described above and seeded into a semi-solid MegaCult-C (Stemcell Technologies, no. 04974) supplemented with the following recombinant human cytokines: with 2.0 U/mL EPO, 10 ng/mL IL-3, 20 ng/mL IL-6, 50 ng/mL SCF, 50 ng/mL thrombopoietin, 20 ng/mL G-CSF, 20 ng/mL M-CSF, and 10 ng/mL GM-CSF (ConnStem). Thirteen days later, colonies were labeled by adding 4 tests per plate of antibodies targeting CD14 (M5E2), CD41a (HIP8), CD66b (6/40c), and CD235a (HI264) (BioLegend 301830, 303704, 392904, 349114) diluted in 300 μL PBS to detect colonies with monocyte, megakaryocyte, granulocyte, and erythrocyte lineages, respectively. Wells were imaged the next day with a Molecular Devices Image Xpress Micro 4 (IXM) high-content microscope to produce high-resolution, whole-well scans at ×10 magnification. For the CFU assay of sorted HSCs, single CD90^+^CD45RA^–^CD34^+^CD38^–^CD45^+^ HSCs were sorted into 384-well plates containing a custom liquid format of the semi-solid media used above by replacing the collagen supplement with 0.12N HCl in dH_2_O. After 14 days, each well was stained by adding the same antibody cocktail as for the semi-solid format diluted in 5 μL PBS per well 24 hours prior to imaging or the flow cytometric readout. For the clonal expansion assay, single HSCs were sorted into 384 wells containing SFEMII medium (Stemcell Technologies) supplemented with 100 ng/mL human stem cell factor (SCF), 100 ng/mL human TPO, 100 ng/mL human FLT3L, 20 ng/mL human IL-6 (cytokines from Peprotech), 0.75 μM StemRegenin 1 (Cayman Chemical), and 500 nM UM729 (Stemcell Technologies) for 7 days. At each experimental endpoint, cells were acquired on a BD LSRFortessa flow cytometer.

### scRNA-seq.

Human Lin^–^CD34^+^ cells were sorted from engrafted bone marrow. Cells were hashed with 0.5 μg TotalSeq anti–human hashing antibody (BioLegend, B0251 for control and B0252 for *DPP9^–/–^*). Approximately 10,000 hashed cells were encapsulated into droplets using the 10x Chromium GEM platform. scRNA-seq libraries were generated using the Chromium Next GEM Single Cell 3′ Reagent Kits version 3.1 (10x Genomics) according to the manufacturer’s instructions and sequenced on an Illumina NovaSeq system. Raw sequencing data were processed using Cell Ranger (version 7.1.0) and aligned to the GRCh38-3.0.0 reference genome. The resulting filtered gene-barcode matrices were imported into R (version 4.2.3) and analyzed using Seurat (version 5.0.1). WT and KO cells were demultiplexed based on hashing antibody signals using the HTODemux() function. Cells were then filtered to exclude those with mitochondrial gene expression of greater than 25%, followed by data normalization, scaling using ScaleData(), and principal component analysis (PCA). Datasets were subsequently merged, clustered, and visualized using UMAP. Differential expression was performed using Seurat’s FindMarkers function. Pathway enrichment analysis was performed using Ingenuity Pathway Analysis (Qiagen).

### Statistics.

Data are shown as the mean ± SD, unless otherwise specified. Statistical significance was determined by 2-tailed Student’s *t* test, or 2-way ANOVA using GraphPad Prism 9.0 (GraphPad Software). A *P* value of less than 0.05 was considered statistically significant. In some figures, relative cell numbers were used instead of absolute numbers to normalize the cell number variations between different tissue donors. The relative cell number is given as the fold change of each individual animal relative to the mean of the control group within each experimental cohort.

### Study approval.

All procedures were carried out following protocols approved by the Yale IACUC. This study is not considered human Subjects research.

### Data availability.

Values for all data points in graphs are included in the Supporting Data Values file. scRNA-seq data generated in this study (GSE332722) have been deposited in the Gene Expression Omnibus (GEO) database. All data needed to evaluate the conclusions in this work are present in the main text or supplemental materials.

## Author contributions

The project was conceptualized by TX, JRB, and AH. TX, JRB, M Carlino, AH, YT, CYL, FZ, M Chen, and HNB performed experiments. QW, LS, and ES provided key reagents. KB, DSK, and RAF provided funding. RAF provided supervision of entire project. TX wrote the original manuscript with substantial input from AHN, and the manuscript was edited by all authors.

## Conflict of interest

The authors have declared that no conflict of interest exists.

## Funding support

This work is the result of NIH funding, in whole or in part, and is subject to the NIH Public Access Policy. Through acceptance of this federal funding, the NIH has been given a right to make the work publicly available in PubMed Central.

- NIH grant R21AI178249 and the Howard Hughes Medical Institute (to RAF).
- American Cancer Society grant PF-25-1435117-01-PFCBI (to TX).
- National Institute of Diabetes and Digestive and Kidney Diseases (NIDDK), NIH grant U54DK106857 (to DSK).
- Yale University Reproductive Sciences Biobank HIC no. 12696, a component of the Department of Obstetrics, Gynecology and Reproductive Sciences, Yale School of Medicine.
- Yale Flow Cytometry Core is supported in part by an NCI Cancer Center Support grant NIH P30 CA016359 (to the Yale Flow Cytometry Core). The BD symphony is supported by a NIH shared instrument grant S10 OD026996.

## Supplementary Material

Supplemental data

Supplemental table 1

Supporting data values

## Version 1 09/15/2026

Electronic publication
