# Supplementary material for: Reverse genetics in humanized mice reveals CARD8-mediated pyroptosis causing pancytopenia in human DPP9 deficiency
Source: J Clin Invest. 2026 Sep 15;136(18):e207530. doi: 10.1172/JCI207530 (PMC13574144; doi:10.1172/JCI207530)
Supplement: Supplementary file 1 [file jci-136-207530-s202.pdf]

Supp Fig 1

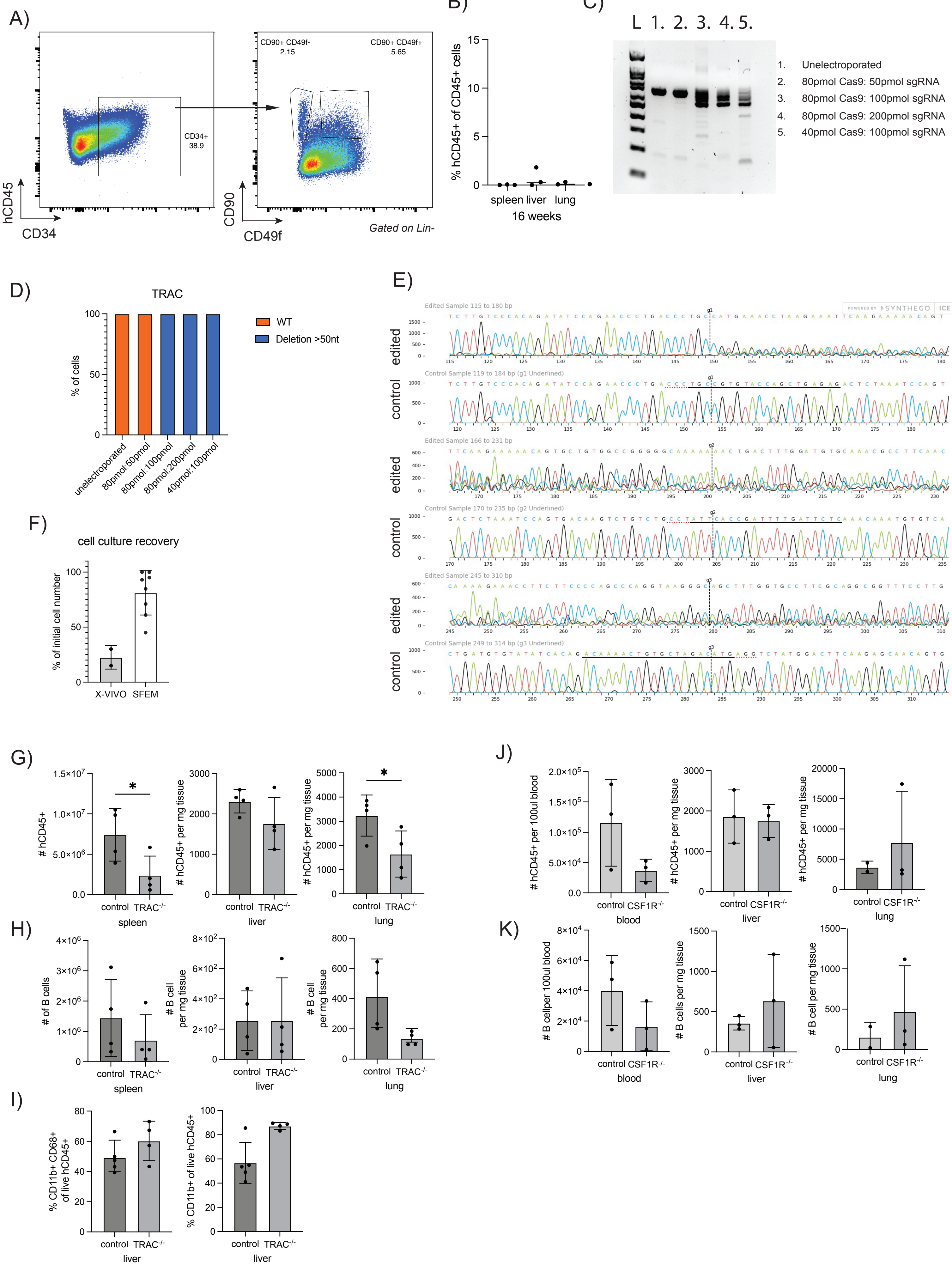
A)
CD90+ CD49f-
2.15
CD90+ CD49f+
5.65
CD34+
38.9
hCD45
CD34
CD90
CD49f
Gated on Lin-
B)
% hCD45+ of CD45+ cells
spleen liver lung
16 weeks
C)
L 1. 2. 3. 4. 5.
1. Unelectroporated
2. 80pmol Cas9: 50pmol sgRNA
3. 80pmol Cas9: 100pmol sgRNA
4. 80pmol Cas9: 200pmol sgRNA
5. 40pmol Cas9: 100pmol sgRNA
D)
TRAC
WT
Deletion >50nt
% of cells
unelectroporated
80pmol:50pmol
80pmol:100pmol
80pmol:200pmol
40pmol:100pmol
E)
edited
control
Edited Sample 115 to 180 bp
Control Sample 119 to 184 bp (g1 Underlined)
Edited Sample 166 to 231 bp
Control Sample 170 to 235 bp (g2 Underlined)
Edited Sample 245 to 310 bp
Control Sample 249 to 314 bp (g3 Underlined)
F)
cell culture recovery
% of initial cell number
X-VIVO
SFEM
G)
# hCD45+
# hCD45+ per mg tissue
control TRAC-/-
spleen
liver
lung
H)
# of B cells
# B cell per mg tissue
control TRAC-/-
spleen
liver
lung
I)
% CD11b+ CD68+ of live hCD45+
% CD11b+ of live hCD45+
control TRAC-/-
liver
J)
# hCD45+ per 100ul blood
# hCD45+ per mg tissue
control CSF1R-/-
blood
liver
lung
K)
# B cellper 100ul blood
# B cells per mg tissue
# B cell per mg tissue
control CSF1R-/-
blood
liver
lung

Supp Fig. 1. (**A**) Markers of iPSCs following hematopoietic stem cell differentiation following manufacturer's protocol (Stemcell technologies). (**B**) Neonatal engraftment of iPSC-derived hematopoietic stem cells in MISTRG6 mice. $hCD45^+$, human $CD45^+$. (**C**) primary CD34+ human HSPCs are electroporated with varying quantity of Cas9 and sgRNA against the TRAC locus. PCR amplicon around the edited region. L: Ladder. (**D**) Inferred Editing Efficacy (Synthego) based on Sanger traces from samples in panel C). (**E**) Sanger traces around the *TRAC* locus around sgRNA 1 cut site (g1), sgRNA 2 cut site (g2), sgRNA 3 cut site (g3). (**F**) $CD34^+$ human HSPCs are cultured overnight in X-VIVO 15 or SFEM media and the number of cells recovered the next day is quantified. (**G**) number of human CD45+ cells, (**H**) human B cells, and (**I**) frequency of human myeloid cells 12-16 weeks after engraftment with control or $TRAC^{-/-}$ CD34+ HSPCs in MISTRG6 mice. (**J**) number of human CD45+ cells and (**K**) human B cells 9 weeks after engraftment with control or $CSF1R^{-/-}$ CD34+ HSPCs in MISTRG6 mice.

Supp Fig 2

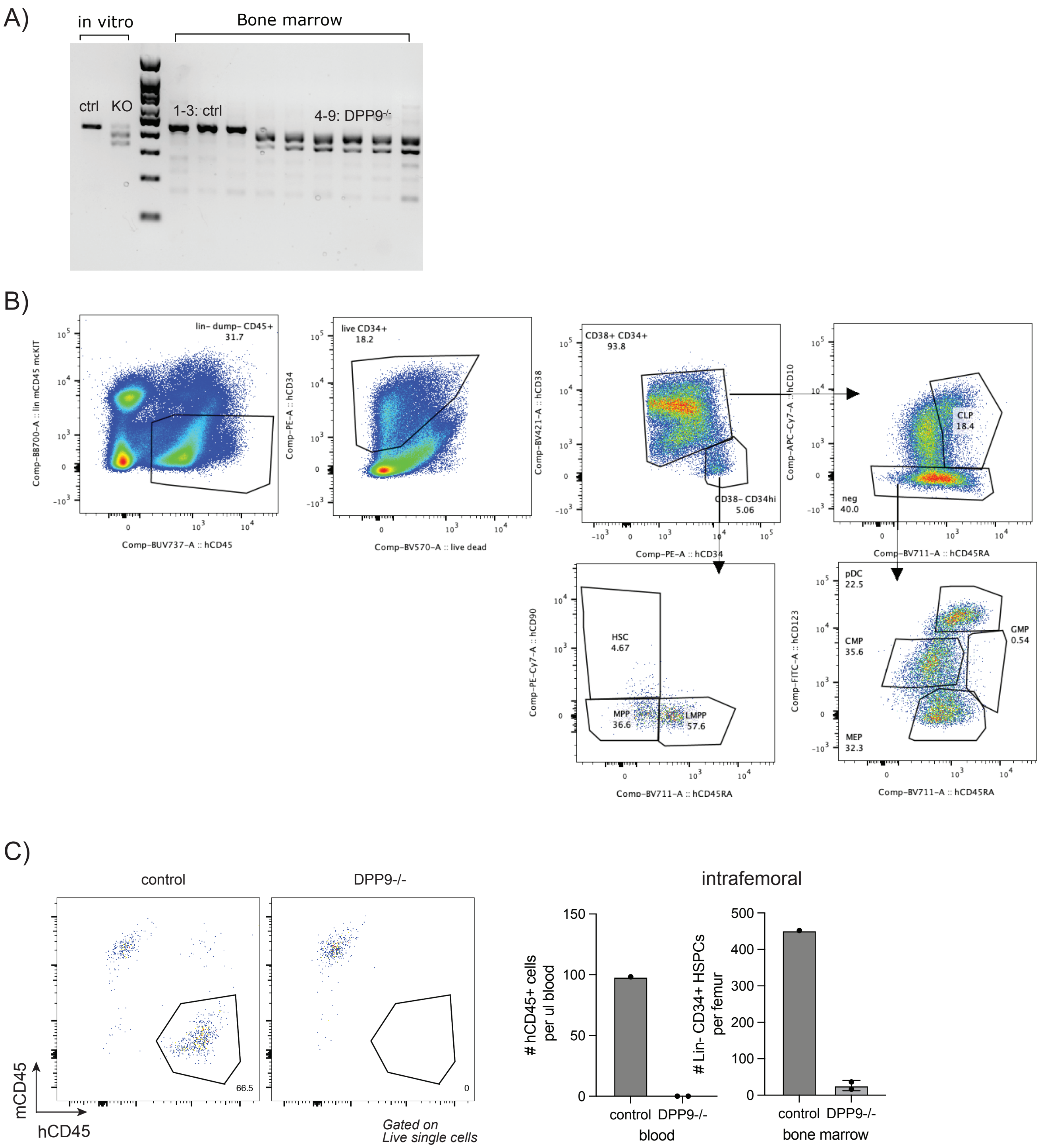


Supp Fig. 2. (**A**) PCR amplification of the DPP9 sgRNA targeting locus. Edited cells have a smaller amplified band due to truncation deletion caused by CRISPR editing. CD34+ cells are either engrafted in vivo or kept in culture for 3 days. DNA from bone marrow 9 weeks after engraftment. (**B**) Gating strategy for bone marrow hematopoietic stem and progenitor populations. (**C**) Control or *DPP9-/-* cells are injected intrafemorally for 16 weeks, cell numbers are assessed in the blood and bone marrow.

Supp Fig 3

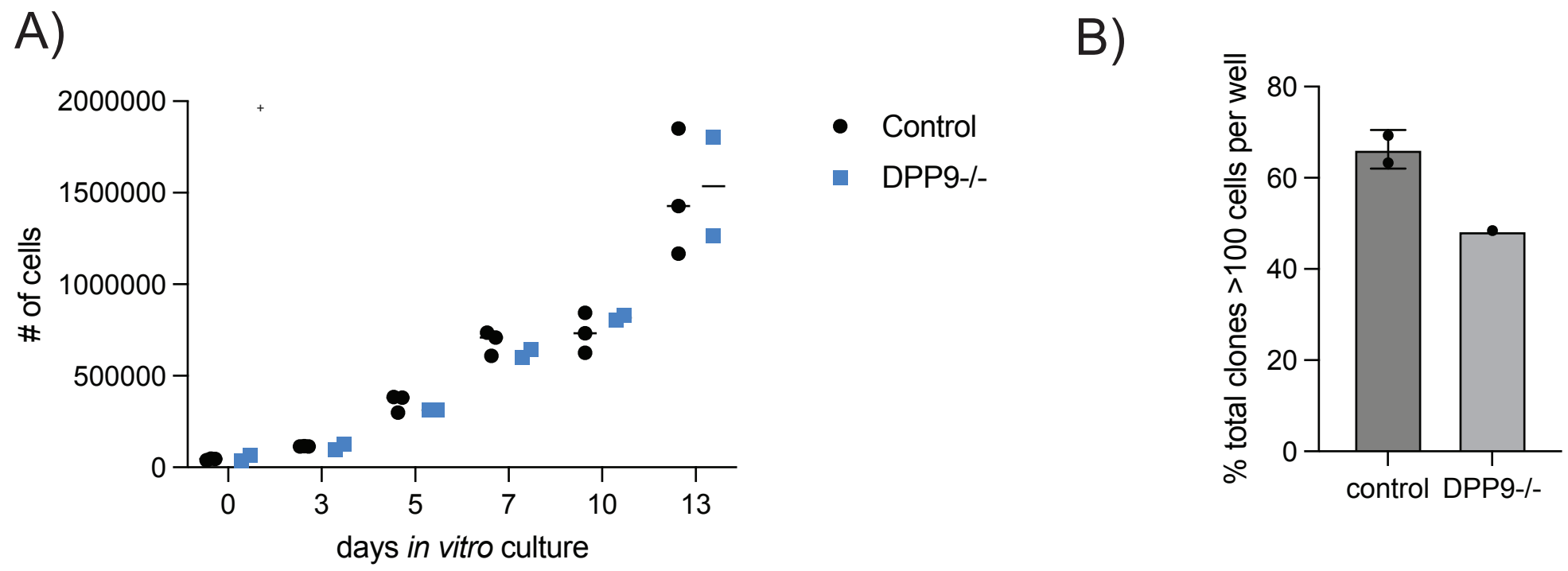


Supp Fig. 3. (**A**) In vitro expansion culture of control and *DPP9-/-* CD34+ HSPCs. (**B**) Frequency of clones that expanded to more than 100 cells at the end of a 7-day expansion of HSCs following experimental scheme in Figure 3D.

Supp Fig 4

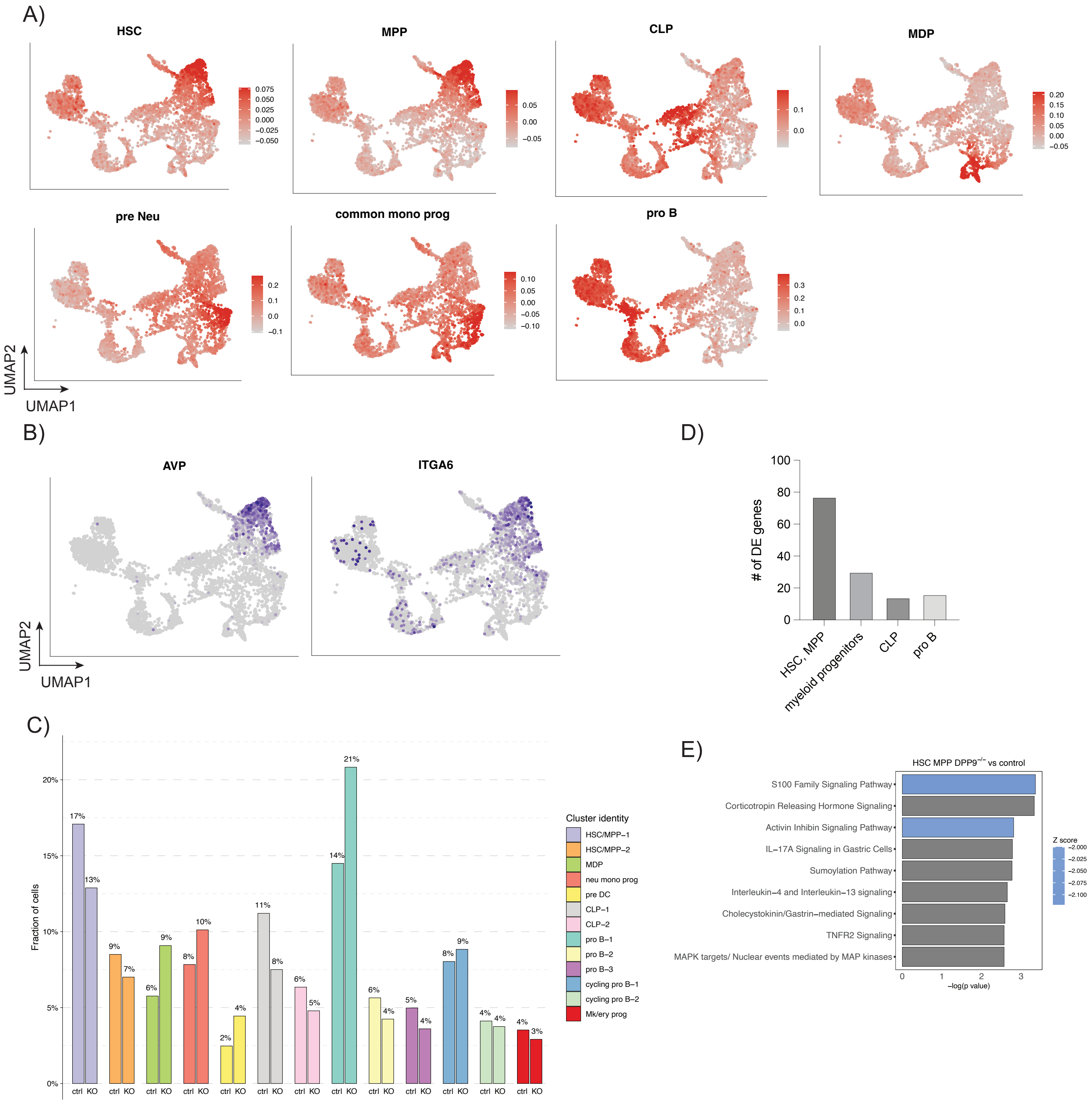
A)
HSC
MPP
CLP
MDP
pre Neu
common mono prog
pro B
UMAP2
UMAP1
B)
AVP
ITGA6
UMAP2
UMAP1
C)
Fraction of cells
0%
5%
10%
15%
20%
17%
13%
9%
7%
6%
9%
8%
10%
2%
4%
11%
8%
6%
5%
14%
21%
6%
4%
5%
4%
8%
9%
4%
4%
4%
3%
ctrl KO
Cluster identity
HSC/MPP−1
HSC/MPP−2
MDP
neu mono prog
pre DC
CLP−1
CLP−2
pro B−1
pro B−2
pro B−3
cycling pro B−1
cycling pro B−2
Mk/ery prog
D)
# of DE genes
0
20
40
60
80
100
HSC, MPP
myeloid progenitors
CLP
pro B
E)
HSC MPP DPP9−/− vs control
S100 Family Signaling Pathway
Corticotropin Releasing Hormone Signaling
Activin Inhibin Signaling Pathway
IL−17A Signaling in Gastric Cells
Sumoylation Pathway
Interleukin−4 and Interleukin−13 signaling
Cholecystokinin/Gastrin−mediated Signaling
TNFR2 Signaling
MAPK targets/ Nuclear events mediated by MAP kinases
−log(p value)
Z score

Supp Fig. 4. (**A**) Overlay of various gene signatures of different human bone marrow populations (49) onto scRNA-seq dataset. (**B**) Expression of *AVP* and *ITGA6* encoding CD49f marking HSC populations. (**C**) Frequency of cells within each cluster for control or *DPP9*$^{-/-}$ cells. WT, control. KO: *DPP9*$^{-/-}$. (**D**) number of differentially expressed (DE) genes defined as FDR <0.1 for each cluster. HSC, MPP includes HSC/MPP-1 and HSC/MPP-2, myeloid progenitors include MDP, neu mono prog, pre DC. CLP includes CLP-1, CLP-2. Pro B includes pro B-1, pro B-2, pro B-3. (**E**)Ingenuity Pathway Analysis of differentially expressed genes found between *DPP9*$^{-/-}$ and control HSC/MPP clusters, DE genes defined as FDR <0.1.

Supp Fig 5

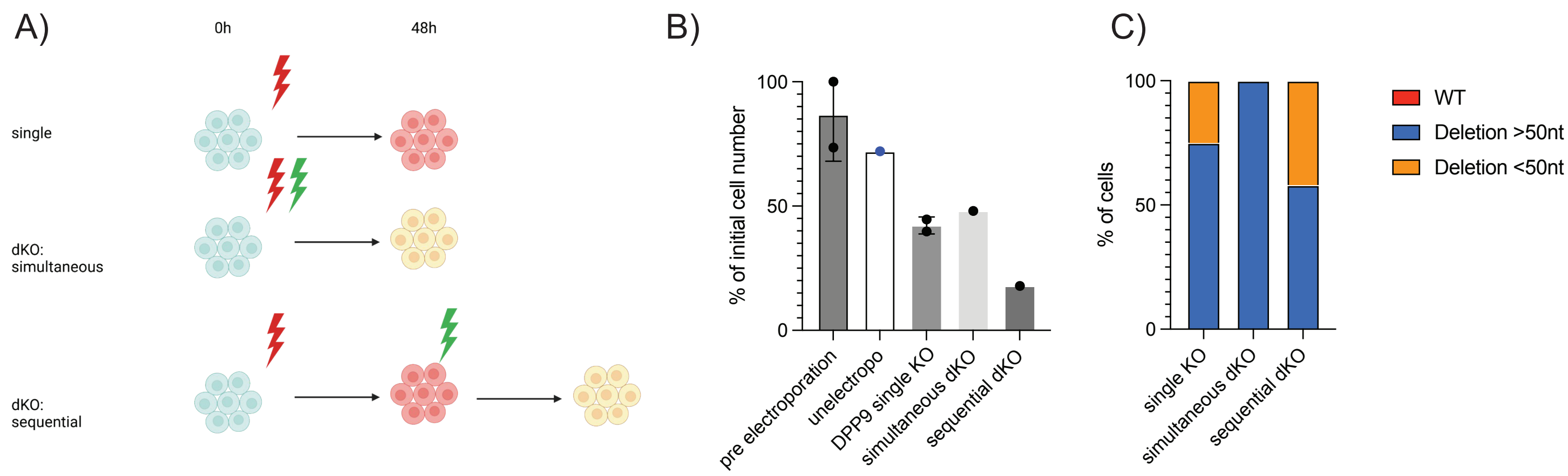


Supp Fig. 5. (**A**) Schematic for sequential or simultaneous double KO. (**B**) Frequency of initial cell number after electroporation. (**C**) gene knockout efficiency of *DPP9* by Sanger sequencing.
